# RNA sequencing-mediated transcriptome analysis of rice plants in endoplasmic reticulum stress conditions

**DOI:** 10.1186/1471-2229-14-101

**Published:** 2014-04-18

**Authors:** Yuhya Wakasa, Youko Oono, Takayuki Yazawa, Shimpei Hayashi, Kenjirou Ozawa, Hirokazu Handa, Takashi Matsumoto, Fumio Takaiwa

**Affiliations:** 1 Functional Transgenic Crops Research Unit, Genetically Modified Organism Research Center, National Institute of Agrobiological Sciences, Kannondai 2-1-2, Tsukuba, Ibaraki 305-8602, Japan; 2 Agrogenomics Research Center, National Institute of Agrobiological Sciences, 2-1-2 Kannondai, Tsukuba, Ibaraki 305-8602, Japan; 3 Government and Public Corporation Information Systems, Hitachi Co., Ltd, 2-4-18 Toyo, Koto-ku, Tokyo 135-8633, Japan

**Keywords:** Gene targeting, ER stress response, Microarray, *Oryza sativa* L, RNA sequencing, Transcriptome

## Abstract

**Background:**

The endoplasmic reticulum (ER) stress response is widely known to function in eukaryotes to maintain the homeostasis of the ER when unfolded or misfolded proteins are overloaded in the ER. To understand the molecular mechanisms of the ER stress response in rice (*Oryza sativa* L.), we previously analyzed the expression profile of stably transformed rice in which an ER stress sensor/transducer *OsIRE1* was knocked-down, using the combination of preliminary microarray and quantitative RT-PCR. In this study, to obtain more detailed expression profiles of genes involved in the initial stages of the ER stress response in rice, we performed RNA sequencing of wild-type and transgenic rice plants produced by homologous recombination in which endogenous genomic *OsIRE1* was replaced by missense alleles defective in ribonuclease activity.

**Results:**

At least 38,076 transcripts were investigated by RNA sequencing, 380 of which responded to ER stress at a statistically significant level (195 were upregulated and 185 were downregulated). Furthermore, we successfully identified 17 genes from the set of 380 ER stress-responsive genes that were not included in the probe set of the currently available microarray chip in rice. Notably, three of these 17 genes were non-annotated genes, even in the latest version of the Rice Annotation Project Data Base (RAP-DB, version IRGSP-1.0).

**Conclusions:**

Therefore, RNA sequencing-mediated expression profiling provided valuable information about the ER stress response in rice plants and led to the discovery of new genes related to ER stress.

## Background

The endoplasmic reticulum (ER) is an organelle in which the synthesis of secretory proteins and the folding and assembly of newly synthesized premature proteins occurs. When these functions are perturbed by the accumulation of unfolded or misfolded proteins in the ER, the cells incur ER stress conditions. ER stress then induces countermeasures in cells referred to as the ER stress response. The ER stress response is a mechanism that maintains ER homeostasis by balancing the folding capacity and folding demands imposed on the ER through the induction of genes encoding chaperones and protein folding-related enzymes, the attenuation of translation, ER-associated degradation, or regulated IRE1-dependent decay (RIDD) [1-3]. Severe ER stress in cells ultimately induces programmed cell death. The mechanisms of the ER stress response are conserved in eukaryotes such as yeast, mammals and plants.

The ER stress response comprises several signaling pathways. In animals, ER stress is sensed by the bZIP-type transcription factor ATF6, a transmembrane protein activated by ER stress-mediated proteolysis via site 1 and 2 (S1P and S2P) proteases [4]. PERK (protein kinase-like ER kinase), a transmembrane kinase, phosphorylates the translation initiation factor eIF2a, resulting in a reduction of protein synthesis and the loading of proteins entering the ER [5]. In rice, OsbZIP39 and OsbZIP60 may be regulated in a similar manner to that of ATF6, as truncated recombinant proteins lacking the C-terminal putative transmembrane domain (TMD) induce the ER stress response [6,7]. Furthermore, the membrane-associated bZIP-type transcription factors AtbZIP17 and AtbZIP28 in *Arabidopsis* also play key roles in inducing the ER stress response [8-10]. AtbZIP17 and AtbZIP28 are also similar to ATF6 in terms of structure and mode of action [10,11]. On the other hand, counterparts of PERK have not been identified in plants.

IRE1, an ER stress sensor protein, is highly conserved in eukaryotes, yeast, mammals and plants. IRE1 is a transmembrane protein that has a kinase domain and a ribonuclease domain in its C-terminal cytosolic region. Accumulation of unfolded or misfolded proteins in the ER lumen induces dimerization of IRE1, autophosphorylation of the kinase domain and activation of the RNase domain [1,2]. Activated IRE1 is implicated in the unconventional cytoplasmic splicing of mRNA encoding key transcription factors. Substrates of the RNase activity of IRE1 in yeast, mammals, *Arabidopsis* and rice (*Oryza sativa* L.) include mRNA encoding the HAC1, XBP1, AtbZIP60 and OsbZIP50 bZIP-type transcription factors, respectively [6,12-17]. The splicing of these mRNAs results in the appearance of an activation domain (yeast and mammals) or a nuclear localization signal (rice) through frame-shift of their translation products [6,12-15]. Recently, RIDD of mRNAs was reported as a new type of ER stress response in mammals, *Drosophila* and *Arabidopsis.* In RIDD, IRE1 mediates the degradation of mRNAs encoding proteins that traverse the secretory pathway under ER stress conditions [18-20]. In rice, we previously reported that RIDD may cause a reduction in the number of mRNAs encoding secretory proteins in ER-stressed cells [21,22].

We recently generated transgenic rice plants in which the single copy of genomic *OsIRE1* was replaced by two types of missense alleles by homologous recombination, leading to a deficiency of the kinase or ribonuclease (RNase) activity of OsIRE1 [22]. This result was achieved by amino acid substitution of the essential Lys residue of the kinase or RNase domains with Ala (producing K519A and K833A, respectively). Homozygous transgenic rice lines of K519A are not viable, whereas homozygous K833A lines exhibit normal vegetative growth in normal growth conditions (without ER stress inducer) in spite of the loss of RNase activity (K833A OsIRE1 is lost the most of its activity for unconventional splicing of *OsbZIP50* mRNA), suggesting that the kinase activity of OsIRE1 plays a vital role that is independent of its ribonuclease activity. On the other hand, in *Arabidopsis*, double T-DNA insertion mutant of *AtIRE1a* and *AtIRE1b* is viable, although this is more sensitive to ER stress treatment than the wild type [17,20]. Therefore, OsIRE1 may have some unique characteristics that arose through the evolutionary process. We previously performed a DNA microarray screen for OsIRE1-dependent genes using OsIRE1 knocked down rice plants in ER stress condition [6,21]. However, the experiment was preliminary and did not covered whole ER stress responsive genes.

In this study, we used RNA sequencing as a tool to obtain detailed expression profiles of genes involved in the initial step of the ER stress response in rice plants. Microarray analysis is commonly used as a tool for transcriptome analysis. This technique has provided important information regarding the gene expression profiles of various biological species. However, although much valuable information has been obtained in rice by microarray analysis using the Agilent 44 K microarray chip [23-27], it remains possible that probes coding for some unidentified mRNAs and non-coding RNAs may not have been included in this chip. On the other hand, as data obtained from RNA sequencing analysis can theoretically cover the complete transcriptome, data from RNA sequencing is expected to complement and extend the current microarray data. Thus, based on RNA sequencing-mediated gene expression analysis, we performed a comparison of comprehensive expression profiles of wild-type and K833A rice plants under ER stress conditions. Using this RNA sequencing technique, we identified novel ER stress response-related transcripts.

## Results and discussion

### Comprehensive screening of ER stress-responsive genes

To obtain detailed information about the expression profiles of genes involved in the ER stress response in root tissues of rice seedlings, especially during the initial stages of the ER stress response, we compared the expression profiles of plants in three pairs of treatment groups: (1) wild type without tunicamycin (TM, an inhibitor of protein glycosylation used as an ER stress-response inducer) treatment vs. wild type with a short period (2 hr) of TM treatment; (2) wild type with DMSO (solvent only) vs. wild type with TM treatment; and (3) wild type with TM treatment vs. K833A with TM treatment. In addition, we constructed cDNA libraries from plants in five different treatment groups (wild type, wild type with TM treatment, wild type with DMSO, K833A with DMSO, and K833A with TM treatment) and sequenced 100 bp paired-end (PE) reads from the libraries using Illumina RNA-Seq technology. A total of 87.0%-92.5% of the total Illumina reads aligned to the IRGSP-1.0 reference rice genome (http://rapdb.dna.affrc.go.jp) sequence, while 57.9–62.7% aligned uniquely to the rice genome (Exonic junction), 27.5–28.8% represented unique junctions (spliced junctions), and 1.5–1.6% returned multiple hits to the genome. Approximately 319 million quality evaluated reads aligned to the rice genome and were used for further analysis (Table 1). We estimated that the expression of 38,076 genes was investigated in this RNA sequencing analysis (as shown in Additional file 1: Figure S1), which covered the entire rice transcriptome, based on its calculated size. Thus, considering that 30,000 genes can be investigated using the currently available Rice 44 K microarray (Agilent Technologies) [28], RNA sequencing data are expected to be far superior to data provided from microarray analysis. In addition, RNA sequencing is not limited to the detection of transcripts that correspond to annotated genes, allowing the identification of new genes.

**Table 1:** Mapping of RNA Seq reads obtained from root of the rice seedlings to the reference IRGSP-1.0 genome sequence

**RNA Seq library**		**Preprocessed**	**Aligned**				**Unaligned**	
			**Exonic regions**	**Spliced junctions**	**Multi**	**%**	**Unaligned**	**%**
WT	No treatment	80,603,572	46,955,002	22,412,248	1,305,712	87.68	9,930,610	12.32
	DMSO	43,228,304	25,033,109	11,899,715	664,838	86.97	5,630,642	13.03
	TM	83,562,536	49,163,467	24,068,823	1,292,484	89.18	9,037,762	10.82
K833A	DMSO	79,804,752	50,036,243	22,555,675	1,217,130	92.49	5,995,704	7.51
	TM	68,584,520	42,516,335	19,163,959	1,009,778	91.41	5,894,448	8.59

First, we identified genes from the expression profile data with expression levels that were statistically altered by ER stress. To eliminate the effect of solvent (DMSO) on the expression profile data as much as possible, data from both the wild type vs. wild type with TM treatment, and the wild type with DMSO only vs. wild type with TM treatment, were used for this analysis. While only three genes were differentially expressed in response to DMSO in the wild type, robust changes in gene expression in response to TM were observed under our experimental conditions (Additional file 2: Table S1). Thus, as DMSO has little effect on the transcriptome of the root tissue of rice seedlings, accurate data regarding the ER stress response could be obtained from the comparison of the wild type with DMSO vs. wild type with TM treatment groups in the present study.

We identified 374 ER stress-responsive transcripts that were unique matches to the sense sequences in The Rice Annotation Project Database (RAP-DB, http://rapdb.dna.affrc.go.jp) updated on April 24, 2013 (version IRGSP-1.0) and exhibited statistically significant changes in gene expression in response to ER stress. Furthermore, we identified six ER stress-responsive transcripts that were not annotated by RAP-DB. Therefore, a total of 380 (374 annotated and six non-annotated in RAP-DB) ER stress-responsive transcripts were found by the statistical analysis of RNA sequencing data. Gene cluster picked up 380 candidates of ER stress responsive genes, which included a few non-coding RNA as well as normal genes encoding protein. Among these transcripts, the expression of 195 genes was upregulated by TM treatment, whereas the remaining 185 genes were downregulated by this treatment (Additional file 3: Table S2). Transcripts with expression levels that changed more than 5-fold (>5.0 and <0.2) in response to TM treatment are shown in Table 2. Further, same data are also shown as heat map (Additional file 4: Figure S2). The profiles of ER stress-responsive genes obtained from RNA sequencing were quite similar to data that we previously obtained using microarray analysis, especially the upregulation of protein folding-related genes such as binding protein (BiP), heat shock protein (HSP)70, protein disulfide isomerase-like (PDIL), ER oxidoreductase 1 (ERO1), and calnexin [6,29,30].

**Table 2:** Characterization of up- (>5-fold) or downregulated (<0.2-fold) transcripts annotated by RAP-DB under ER stress condition

	**Gene**	**Description**	**FPKM**		**Fold change**	**FDR**
			**DMSO**	**TM**		
> 5	Os05g0428600	OsBiP4	1.10	94.81	86.36	0
	Os05g0367800	OsBiP3	1.00	28.81	28.81	8.51224E-40
	Os09g0512700	Fes1-like	14.51	347.38	23.94	0
	Os03g0710500	OsBiP2	1.03	16.95	16.45	0.000805324
	Os03g0832200	Calreticulin	12.20	162.11	13.29	0
	Os05g0591400	HSP70	1.08	12.33	11.36	0.000529672
	Os07g0593400	Golgi transport 1 protein B	2.95	33.29	11.30	3.71682E-06
	Os03g0293000	DnaJ domain containing protein	15.75	167.48	10.63	0
	Os08g0156100	Conserved hypothetical protein	4.91	43.59	8.89	7.11559E-08
	Os06g0622700	OsbZIP50	27.89	237.66	8.52	0
	Os06g0593100	UDP-galactose/UDP-glucose transporter	46.56	343.21	7.37	0
	Os04g0670500	Cysteine protease 1 precursor	1.17	8.60	7.33	2.30013E-05
	Os09g0451500	OsPDIL2-3	81.99	562.22	6.86	0
	Os06g0697500	ATPase	1.82	12.27	6.74	1.02548E-06
	Os02g0584700	Heavy metal transport/detoxification protein	4.79	28.97	6.04	5.35941E-05
	Os08g0156000	Conserved hypothetical protein	29.55	175.40	5.94	0
	Os05g0187800	Similar to Derlin-1	51.68	297.54	5.76	0
	Os06g0212900	HSP70	1.46	8.28	5.68	3.75258E-05
	Os03g0733800	Ero1	18.07	101.20	5.60	0
	Os01g0517900	HSP70	1.00	5.48	5.48	1.41916E-10
	Os01g0517850	Luminal-binding protein	1.00	5.48	5.48	1.41916E-10
	Os01g0280500	Eukaryotic translation initiation factor 6	3.30	17.99	5.45	1.62648E-05
	Os03g0820300	ZPT2-14	6.31	34.12	5.41	1.17726E-06
	Os07g0661100	Glycosyl transferase	24.66	130.99	5.31	0
	Os01g0510200	Conserved hypothetical protein	33.02	169.75	5.14	4.85665E-12
	Os02g0115900	BiP1	138.93	710.22	5.11	0
	Os08g0278900	SDF2-like	29.90	151.76	5.08	9.33971E-12
	Os02g0115950	Glutamate dehydrogenase	127.05	644.35	5.07	9.40366E-07
	Os07g0605800	STF-1	7.44	37.60	5.05	9.14091E-10
< 0.2	Os11g0539200	Xyloglucan endotransglycosylase XET2	49.84	6.74	0.135	0
	Os07g0432333	Thionin-like peptide	24.74	3.89	0.157	2.98571E-06
	Os02g0268050	Expansin-A23	6.07	1.00	0.165	0.0369366

**Figure 1:**
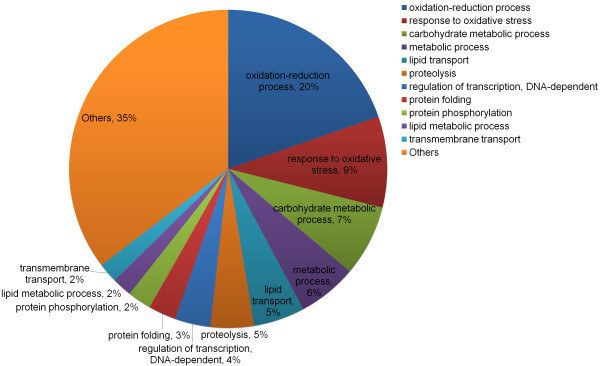
Distribution of Gene Ontology categories (biological processes) for ER stress-responsive transcripts.

We used Gene Ontology (GO) classification to assign the functional categories of RAP-annotated, TM-responsive transcripts using GO terms in the biological process category (Figure 1). These categories included transcripts for the oxidation-reduction process (GO:0055114), which respond to oxidative stress (GO:0006979) in a manner similar to that of the general stress response. Transcripts for proteolysis (GO:0006508), protein folding (GO:0006457) and transmembrane transport (GO:0055085) were also detected, which clearly validated the RNA-Seq expression profiling data obtained from rice tissue under ER stress conditions.

**Figure 2:**
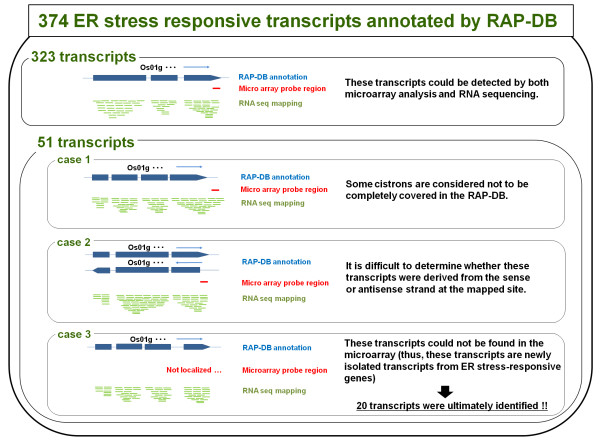
**Process used to identify novel ER stress-responsive transcripts from 374 candidate transcripts in which differential expression was annotated by RAP-DB.** The term ‘novel ER stress-responsive transcript’ is defined as a transcript whose expression could not be detected by microarray analysis. RAP ID numbers, their annotation regions on the rice genome, probe sequences on the microarray chip and mapping data of RNA sequencing were determined by visual observation. Blue arrows indicate the direction of the transcript (5′ to 3′).

**Figure 3:**
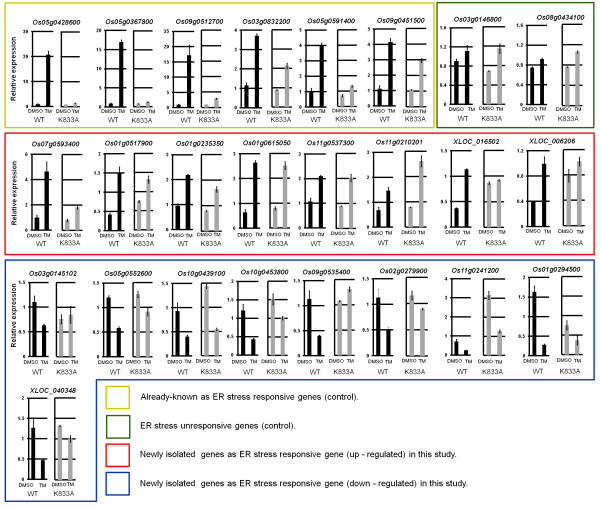
**Quantitative real-time RT-PCR analysis of candidate of novel ER stress-responsive genes.** Three independent rice plants without TM (DMSO) treatment and with TM treatment were analyzed in wild type (black bars) and K833A (grey bars). Some control genes such as already known ER stress-responsive genes (yellow enclosure) and ER stress unresponsive genes (green enclosure) are also shown. A red enclosure indicates newly isolated genes as ER stress responsive gene (up - regulated) in this study. A blue enclosure indicates newly isolated genes as ER stress responsive gene (down - regulated) in this study.

Among the 374 ER stress-responsive transcripts annotated by RAP-DB, we identified 51 transcripts (29 upregulated and 22 downregulated) that were not contained in the probe set on the Rice 44 K microarray chip. Therefore, we examined the relationship among the RAP ID numbers of these 51 transcripts, as well as their annotation regions, probe sequences on microarray chips, and mapping data from RNA sequencing using visual observation (Figure 2). These 51 transcripts were divided into the following three categories: (1) the array probe sequence is localized outside of the cistron predicted by RAP-DB, but the mapping data from RNA sequencing includes the region of the array probe sequence (i.e., some cistrons are considered not to be completely covered in the RAP-DB; Figure 3, case 1); (2) the transcript is predicted to represent both the sense and antisense strands for one locus (i.e., the array probe could not detect gene expression from both strands; Figure 3, case 2); and (3) the array probe could not detect the transcript (i.e., the transcript is a newly isolated transcript from an ER stress-responsive gene; Figure 3, case 3). It should be noted that in the RNA sequencing method that we utilized (refer to Methods), cDNA libraries were primarily produced and were sequenced using an Illumina High-Seq 2000. Thus, for case 2 transcripts, it was difficult to determine whether the transcript was derived from the sense or antisense strand at the mapped site unless the positions of exons and introns were quite different between the sense and antisense transcripts. Therefore, we removed the transcripts corresponding to case 2 from the group of candidates for ER stress-responsive genes. As a result, 20 of the 51 transcripts were ultimately considered to be candidates for ER stress-responsive transcripts (including ten upregulated transcripts and ten downregulated transcripts; Table 3 and Figure 2). Additionally, Table 3 is replaced with heat map data in Additional file 5: Figure S3. On the other hand, six transcripts were identified as ER stress-responsive transcripts that were not annotated in the RAP-DB. The RNA sequencing mapping pattern of these transcripts on the rice genome showed that five of the six transcripts were apparently derived from genes. Four transcripts were clearly upregulated by ER stress and the rest were downregulated. One of the five transcripts was approximately 12 kb long. This gene has an approximately 1.6 kb long terminal repeat (LTR) at both the 5′ and 3′ ends and includes domains encoding reverse transcriptase and ribonuclease H (RNase H) in its internal region. At least ten copies of similar sequences are interspersed in the rice genome. These features are typically observed in copia-like class retrotransposable elements [31]. Although this retrotransposable element is present in multiple copies with high homology in the rice genome, only one locus (chr05:15731011–15742930) exhibited an altered mapping pattern under ER stress conditions. Since this sequence has some clear characteristics of retrotransposable elements, further analysis of the relationship between this sequence and ER stress will be performed in the near future. The remaining 25 transcripts (including 20 annotated and five non-annotated transcripts in the RAP-DB) were identified as novel ER stress-responsive transcripts from RNA sequencing data, the RAP-DB and probe information of microarray (Table 3). These 25 transcripts could not have been identified without the use of RNA sequencing analysis.

**Table 3:** New ER stress responsive transcripts identified by Illumina sequencing

**RAP or gene ID**	**Description**	**FPKM**		**Fold change**	**FDR**
		**WT DMSO**	**WT TM**		
Os07g0593400	Golgi transport 1 protein B	2.95	33.29	11.30	3.72E-06
Os01g0517900	HSP70	1.00	5.48	5.48	1.42E-10
Os01g0627967	Hypothetical protein	6.03	29.71	4.93	0.0350306
Os03g0427500	-	3.78	17.98	4.76	0.00393106
Os01g0235350	Conserved hypothetical protein	3.57	14.00	3.92	0.000037847
Os05g0149300	1-aminocyclopropane-1-carboxylate oxidase	8.40	32.94	3.92	0.000047
Os01g0615050	Proteinase inhibitor I13	11.57	43.08	3.72	0.000334385
Os11g0537300	DedA	20.71	69.03	3.33	0.000000236
Os11g0210201	Conserved hypothetical protein	9.39	30.24	3.22	0.0105401
Os08g0391000	Hypothetical protein	16.31	35.79	2.19	0.0307015
Os10g0169400	-	209.74	105.45	0.50	0.0477132
Os03g0145102	Leucine Rich Repeat family protein	9.52	4.77	0.50	0.0280715
Os05g0552600	Root cap periphery gene2	28.11	13.33	0.47	0.00317487
Os10g0439100	cDNA clone:002-180-G03, full insert sequence	12.34	5.57	0.45	0.0453252
Os05g0563550	FAS1 domain containing protein	61.33	27.34	0.45	0.0143764
Os10g0453800	-	58.58	26.09	0.45	0.00837208
Os09g0535400	Curculin-like lectin domain containing protein	13.52	5.77	0.43	0.00820103
Os02g0279900	-	38.25	15.56	0.41	0.000219466
Os11g0241200	Protein of unknown function DUF538 family protein	19.33	7.27	0.38	0.0180555
Os01g0294500	Class III peroxidase 9	11.95	3.54	0.30	0.000289172
XLOC_016502	chr03:28644979-28645849	1.00	3.27	3.27	0.00163845
XLOC_006206	chr01:34084695–34086611, Cytochrome P450	1.21	3.32	2.75	0.0152738
XLOC_031744	chr07:24188274–24189220, HGWP motif containing protein	1.00	2.22	2.22	0.0342339
XLOC_021400	chr05:15731011–15742930, *copia* type retrotransposable element	1.62	3.57	2.21	1.71595E-05
XLOC_040348	chr10:16566508-16567867	32.17	12.84	0.40	7.41391E-05

Transcripts named by Os number are anotated by RAP-DB. The others (XLOC・・) are non-anotated transcripts.

Then, we performed quantitative real-time RT-PCR (qRT-PCR) analysis to determine whether these 25 genes exhibited the expected changes in expression under the same ER stress conditions used for RNA-Seq analysis (5 μg/mL TM for 2 hr). As shown in Figure 3, the expression patterns of 17 of the 25 genes were similar to those observed using RNA sequencing. It should be noted that 14 of the genes have already been annotated on the rice genome in the RAP-DB, while the others have not yet been annotated (Figure 3). On the other hand, specific RT-PCR product could not be obtained from the remaining eight genes even under various PCR conditions because it is difficult to design of specific primer. Alternatively, in light of the notion that our data theoretically include all possible transcripts in the rice tissue that was examined, some of the transcripts may have been derived from genes with faint levels of expression, resulting in the failure to produce PCR product by qRT-PCR amplification for these eight genes.

We also investigated expression levels of these 17 genes in K833A line treated or non-treated with TM (Figure 3). In eight ER stress-upregulated genes, *Os07g0593400 Os01g0517900*, *XLOC_006206* and *XLOC_016502* expression showed lower induction by TM than wild type with TM treatment but the other four genes were not observed an effect of K883A mutation on their expression changes under ER stress condition. On the other hand, in nine ER stress-downregulated genes, expression levels of *Os03g0145102*, *Os05g0552600*, *Os09g0535400*, *Os02g0279900* and *XLOC_040348* became less intense their reduction in K833A compared with wild type with TM. The other four genes were not detected clear difference their expression changes between the wild type and K833A (Figure 3).

### The relationship between ER stress-responsive gene and two ER stress-response induction pathways

The induction of ER stress response-related genes in plants is mainly controlled by two pathways, which in rice includes a pathway involving the OsbZIP39 and OsbZIP60 transcription factors, as well as the OsIRE1/OsbZIP50 pathway [6,7], while in *Arabidopsis*, these pathways involve AtbZIP17 and AtbZIP28, and AtbZIP60/AtIRE1. To discuss whether the 195 ER stress-inducible genes (containing eight newly isolated genes as ER stress-upregulated gene) revealed by RNA sequencing are regulated by the former (OsbZIP39 and OsbZIP60) or latter (OsIRE1/OsbZIP50) pathway, we compared the expression patterns of these genes in wild type vs. K833A plants treated with TM. If OsbZIP39, OsbZIP60 and/or unknown factor exclusively induce some gene expressions, fold changes between the wild type with TM and K833A with TM would be shown as similar level. On the contrary, in the case of that OsIRE1/OsbZIP50 pathway exclusively induces some gene expression, their gene expressions would be increased in wild type with TM but be little changed in K833A with TM. If not only OsbZIP39 and OsbZIP60 but also OsIRE1/OsbZIP50 pathway were involved in some gene expression, expression levels would be increased by TM treatment in both of wild type and K833A but their fold changes in wild type must be higher than K833A. However, there may be some exceptional genes. We consider that 195 ER stress-upregulated genes isolated by RNA seq analysis can be categorized into above four patterns.

We predictably observed four different types of expression patterns: (1) genes whose expression was induced by TM treatment in both the wild type and K833A (approximately 15%); (2) genes whose expression in K833A was moderately suppressed by TM treatment (approximately 35%); (3) genes whose expression in K833A was strongly suppressed by TM treatment (approximately 31%); and (4) genes with inconsistent expression patterns (approximately 19%). We postulate that genes exhibiting the type (1) expression pattern are exclusively induced by OsbZIP39 and/or OsbZIP60, but not by OsIRE1/OsbZIP50, whereas genes exhibiting the type (2) expression pattern are controlled by both pathways. Furthermore, perhaps genes exhibiting the type (3) expression pattern are exclusively induced by the OsIRE1/OsbZIP50 pathway under ER stress conditions. Ten representative genes exhibiting each type of expression pattern and their heat map are shown in Table 4 and Additional file 6: Figure S4. Further, original data of the basis of Table 4 is also shown as Additional file 7: Table S3. Incidentally, qRT-PCR analysis suggests that *Os01g0235350*, *Os01g0615050*, *Os11g0537300* and *Os11g0210201* are controlled by OsbZIP39, OsbZIP60 and/or unknown factor rather than OsIRE1/OsbZIP50 pathway. *Os07g0593400*, *Os01g0517900* and *XLOC_006206* would be controlled by not only OsbZIP39, OsbZIP60 and/or unknown factor but also OsIRE1/OsbZIP50 pathway. *XLOC_016502* may be exclusively regulated by OsIRE1/OsbZIP50 pathway (Figure 3). The categorization results of *Os01g0517900*, *Os01g0235350* and *Os11g0537300* were not identical between the RNA seq and qRT-PCR due to slight differences of their fold changes. Because most of genes newly isolated as ER stress responsive gene in this study show a tendency of relative lower level of expression in rice root, experimental error might be conspicuous in these genes.

**Table 4:** Categories of expression changes between the wild type and K833A under ER stress condition (representative 10 genes, respectively)

**RAP ID**	**FPKM**		**Fold change**	**FDR**	**FPKM**		**Fold change**	**FDR**
	**WT**				**K833A**			
	**DMSO**	**TM**			**DMSO**	**TM**		
Expression changes were little influenced by K833A *1								
Os01g0837000	9.08	18.38	2.03	0.00859618	12.09	25.78	2.13	0.000020584
Os03g0300400	109.61	183.59	1.67	0.0467696	186.45	305.11	1.64	0.0134221
Os03g0187800	7.05	15.94	2.26	0.00948828	19.90	50.20	2.52	4.20687E-08
Os05g0427400	29.33	55.84	1.90	0.0300822	77.44	146.93	1.90	0.00251439
Os06g0586000	29.61	66.60	2.25	0.0395364	77.79	164.57	2.12	0.000156006
Os07g0182100	64.47	122.61	1.90	0.00326075	167.04	277.44	1.66	0.023173
Os08g0135900	33.96	65.11	1.92	0.0453252	90.63	164.43	1.81	0.0696732
Os09g0412300	9.26	21.71	2.34	0.0443049	12.18	30.56	2.51	0.000226511
Os09g0571200	25.91	56.86	2.19	0.00289657	51.23	103.80	2.03	0.0000892
Os11g0149400	82.59	150.16	1.82	0.02882	113.98	177.32	1.56	0.0892376
Expression changes were moderately infuluenced by K833A *2								
Os02g0710900	29.26	116.88	3.99	3.45959E-08	31.38	69.09	2.20 0.00549388	
Os02g0115900	138.92	710.21	5.11	0	189.82	443.48	2.34	0.00122459
Os03g0832200	12.19	162.1	13.30	0	9.77	49.78	5.10	0
Os05g0187800	51.68	297.53	5.76	0	62.5	152.53	2.44	1.1548E-07
Os06g0593100	46.55	343.21	7.37	0	61.77	254.62	4.12	0
Os06g0622700	27.89	237.66	8.52	0	48.45	172.85	3.57	0
Os07g0593400	2.94	33.29	11.32	3.71682E-06	2.57	7.65	2.98	0.115152
Os08g0440500	29.9	117.73	3.94	3.45443E-11	36.2	73.04	2.02	0.00135378
Os08g0155900	21.56	81.04	3.76	2.14632E-06	25.68	43.3	1.69	0.192226
Os09g0451500	81.98	562.21	6.86	0	94.77	261.31	2.76	5.00997E-11
Expression changes were drastically infuluenced by K833A *3								
Os01g0947000	10.91	30.5	2.80	3.55683E-06	12.06	13.49	1.12	0.999987
Os03g0710500	1.03	16.95	16.46	0.000805324	1.012	1.016	1.00	1
Os03g0733800	18.07	101.2	5.60	0	24.18	37.53	1.55	0.219289
Os04g0670500	1.17	8.59	7.34	2.30013E-05	1.09	1.49	1.37	1
Os05g0591400	1.08	12.32	11.41	0.000529672	1.017	1.023	1.01	1
Os05g0428600	1.097	94.81	86.43	0	1.018	1.3	1.28	1
Os06g0139800	7.87	20.91	2.66	0.00237693	9.14	10.42	1.14	0.999987
Os06g0212900	1.45	8.28	5.71	3.75258E-05	1.29	1.35	1.05	1
Os07g0123900	88.78	196.86	2.22	5.81855E-05	66.84	75.68	1.13	0.999987
Os12g0568500	77.5	283.8	3.66	2.3146E-10	28.7	25.71	0.90	0.999987

*1 These genes are exclusively controlled by OsbZIP39, OsbZiP60 and/or unknown factor (not controlled by OsIRE1), namely, these genes showed little effect of K833A mutant on gene expression under the ER stress condition. Thus fold change between wt vs. wt with DTT and K833A vs. K833A with DTT showed similar in ‘little’ category genes.

*2 These genes are controlled by not only OsbZiP39, OsbZiP60 and/or unknown factor but also OsIRE1 because these gene expressions are induced by TM treatment in even K833A plant but their expression levels are lower than wild type with TM treatment.

*3 These genes are exclusively controlled by OsIRE1 pathway when ER stress is occurred because these gene expressions are little induced by TM treatment in K833A plant but their expression levels are increased in wild type with TM treatment.

### Expression patterns of candidate RIDD target genes

We previously reported that the transcript levels of some genes were reduced by RIDD-like behavior under ER stress conditions [21,22]. In the current study, 185 genes in the wild type were downregulated by 2 hr of TM treatment. RIDD-like changes in expression were observed in 10 of the 185 genes, i.e., the mRNA levels of these ten genes were not clearly suppressed in the K833A line treated with TM (Additional file 8: Table S4). One of these ten genes, *Os03g0663400*, had already been considered a candidate RIDD target gene in a previous study using microarray-mediated screening [21]. On the other hand, other candidate RIDD target genes (e.g., *Os03g0103100*, *Os05g0477900*, *Os06g0726100*, *Os10g0552600* and *Os11g0645400*) that were characterized in previous studies did not exhibit RIDD-like changes in expression in the current study. On the other hand, although only 10 genes as candidate of RIDD target could be detected by analysis of RNA seq data, *Os03g0145102*, *Os05g0552600*, *Os09g0535400*, *Os02g0279900* and *XLOC_040348* that were newly isolated as stress responsive genes by qRT-PCR analysis between the wild type and K833A may be also candidate RIDD target genes from their expression pattern (Figure 3). Since RIDD-like changes in the expression of these genes had clearly been detected in response to 4 hr of TM treatment or 2 hr of 2 mM DTT treatment in rice root tissues [21,22], perhaps clear RIDD-like behavior of these genes was not detected in the current study because we only used 2 hr of TM treatment. In *Arabidopsis* seedling, 5 μg/L TM treatment for 2 hr is sufficient to induces RIDD response [20]. Sensitivity against ER stress inducer such as TM may be different between the *Arabidopsis* and rice seedling. On the other hand, we initially expected that genome mapping of RNA sequencing data would be able to reveal the initial stages of RIDD since this technique reveals changes in the mapping patterns of genes even if their apparent expression levels are not altered. However, unfortunately, the expected data were not obtained by examining the mapping pattern of RNA sequencing data under our experimental conditions. One possible explanation is that mRNA degradation by RIDD may be quite smooth reaction, so that we failed to detect mRNAs which were partially digested by RNase activity of OsIRE1.

### Orthologous genes of newly identified ER stress-responsive genes in Arabidopsis

We examined whether orthologous sequences of these 17 transcripts exist in the *Arabidopsis* genome by searching the *Arabidopsis* Information Resource (TAIR) database (http://www.arabidopsis.org/index.jsp), and we investigated whether any such genes are also induced by ER stress in *Arabidopsis.* From data reported by Nagashima *et al*. (2011) and Mishiba *et al.* (2013) [17,20], nine transcripts (*Os07g0593400*, *Os01g0517900*, *Os01g0235350*, *Os01g0615050*, *Os11g0537300*, *Os11g0241200*, *Os10g0439100*, *Os05g0552600,* and *XLOC_006206*) were assigned as homologs of genes 0in the *Arabidopsis* genome, but homologs of the other eight transcripts were not detected. Interestingly, *Os01g0517900*, *Os01g0294500* and *Os10g0439100* were reported as ER stress-responsive genes in previous reports [17,20], and the expression patterns of these individual homologous genes in response to the ER stress inducer TM were similar between rice and *Arabidopsis*.

### Prediction of micro (mi)RNA target transcripts in ER stress responsive genes

Recently, miRNA-mediated regulation in ER stress response is reported [32]. Thus we preliminary searched miRNA target from 380 ER stress-responsive transcripts using the search tool ‘psRNATarget’ on web page ‘miRbase database’ (http://plantgrn.noble.org/psRNATarget/) (Additional file 9: Table S5). Fifteen (up-regulation under ER stress condition) and 21 (down-regulation under ER stress condition) genes were predicted as miRNA target (Additional file 9: Table S5). Further experiments need to verify the relationship between the miRNA and these predicted genes.

## Conclusions

In this study, we performed RNA sequencing-mediated transcriptome analysis to elucidate the molecular mechanisms underlying the ER stress response in rice. Novel ER stress-responsive genes that were not detected by microarray chip analysis were identified by RNA sequencing. Furthermore, we also obtained detailed expression profiles of genes involved in the ER stress response by examining a unique disrupted OsIRE1 line (K833A) deficient in RNase activity generated by homologous recombination as well as wild-type plants that were treated with the ER stress inducer TM. The data provide important information regarding the OsIRE1-mediated ER stress response in rice. Furthermore, the RNA sequencing data obtained in this study will help improve the RAP-DB and enhance the development of a new microarray chip in the future.

## Methods

### Plant materials

Non-transgenic rice (*Oryza sativa* L. cv. Nipponbare) and the transgenic rice line K833A, whose *OsIRE1* (*Os07g0471000*) gene was replaced by missense alleles, resulting in a defect in ribonuclease activity, were used in this study [22]. K833A line is seriously defective in the splicing of *OsbZIP50* mRNA under the ER stress condition, thus OsbZiP50 is not available as transcriptional factor in K833A line. On the other hand, the other ER stress-related transcriptional factors, OsbZiP39 and OsbZIP60, are no affected by K833A mutation. The plants were grown on hormone-free solid MS medium (1× Murashige and Skoog salt mixture, 3% sucrose, B5 vitamin, 2.5 mM MES [pH 5.8] and 0.25% gelrite) at 25°C under 16 h light/8 h dark conditions. For ER stress-induction treatment, root tissues of seedlings (7 days after germination) were incubated in liquid MS medium containing 5 μg/L tunicamycin (TM) as an ER stress-inducing reagent for 2 hr at room temperature. For the negative control plants, an equal volume of solvent (DMSO) was added instead of TM. All samples were prepared in triplicate.

### RNA extraction

For all samples, including the wild type, wild type with TM treatment, wild type with solvent (DMSO) only, and K833A with TM treatment samples, total RNA was prepared from root tissues using an RNeasy Plant Mini Kit (Qiagen, Maryland, USA). The RNA was checked for integrity before performing the RNA sequencing process using the Bioanalyzer 2100 algorithm (Agilent Technologies, Tokyo, Japan).

### RNA sequencing

For cDNA library construction, total RNA was extracted from root samples and processed using a TruSeq^TM^ RNA Sample Preparation Kit (Illumina, Tokyo, Japan). Fifteen cDNA libraries were used to generate 319 million PE reads. Sequencing was carried out on each library to generate 100 bp PE reads for transcriptome sequencing on an Illumina High-Seq 2000 platform by a commercial service provider (Takara, Tokyo, Japan).

### Data analysis

Raw sequences in FASTQ format obtained from the Illumina platform were analyzed using publicly available tools. Low-quality bases (Q < 15) were trimmed from both ends of the sequences using a customized program, and the adapters were trimmed using Cutadapt [33] (http://code.google.com/p/cutadapt/). The sequences were mapped to the IRGSP-1.0 reference genome sequence using a series of programs, including Bowtie for short-read mapping [34] and TopHat for defining exon–intron junctions [35]. Reference-based assembly of the reads was performed using Cufflinks and Cuffmerge (http://cufflinks.cbcb.umd.edu/) [36]. The expression level of each transcript was expressed as the fragments per transcript kilobase per million fragments mapped (FPKM) value, which was calculated based on the number of mapped reads. Cuffdiff was used to detect differentially expressed genes using at least two replicates, with a correlation coefficient of >0.90 in each library based on FPKM values (one was added to avoid division by zero when calculating fold changes). A GO term was assigned to each transcript based on the GO annotations for biological process in RAP-DB (The Rice Annotation Project Database [http://rapdb.dna.affrc.go.jp]).

### Quantitative real time RT-PCR (qRT-PCR)

The expression of ER stress responsive genes in root was confirmed by qRT-PCR analysis using three technical replicates from one of the three biological replicates used for RNA-seq analysis. Total RNA was extracted from those samples using the RNeasy Plant Kit (Qiagen, Hilden, Germany) and treated with DNase I (Takara, Shiga, Japan). The first-strand cDNA was synthesized using the Transcriptor First Strand cDNA synthesis kit (Roche, Basel, Switzerland) according to the manufacturer’s protocol. The resulting cDNAs were amplified in the LightCycler^®^ 480 system (Roche, Basel, Switzerland) using transcript-specific primers (Additional file 10: Table S6). The detection threshold cycle for each reaction was normalized using rice *ubiquitin1* with 5′- CCAGGACAAGATGATCTGCC-3′ and 5′-AAGAAGCTGAAGCATCCAGC-3′ as primers. Relative expression levels were calculated with ΔΔCT method.

## Competing interests

The authors declare that they have no competing interests.

## Authors’ contributions

YW and YO contributed equally to this research. YW, YO, TY and SH conducted the experiment. YW, YO and SH drafted the manuscript with edits from KO, HH, TM and FT. All authors read and approved the final manuscript.

## Supplementary Material

Additional file 1: Figure S1 Quantification of gene expression levels by RNA-Seq analysis in rice roots under TM treatment. Scatter plot shows FPKM (Fragments Per Kilobase of transcript per Million fragments sequenced) values of RNA-Seq data from wild type treated with DMSO and WT treated with TM. The abscissa and ordinate show the FPKM of each treatment. Click here for file

Additional file 2: Table S1 Differentially expressed genes between untreated and DMSO-treated samples. Click here for file

Additional file 3: Table S2 List of ER stress-responsive transcripts identified by statistical analysis (upregulated genes in sheet 1, downregulated genes in sheet 2). The expression levels are expressed at the FPKM (Fragments Per Kilobase of transcript per Million fragments sequenced) value. Fold changes (TM/DMSO) were calculated based on FPKM values (see Methods). Click here for file

Additional file 4: Figure S2 Differential gene expression heat map from Table 2. Z scores of RPKM (Reads Per Kilobase of exon Model per million mapped reads) values for each sample were shown in heatmap. The bar in red-black gradation indicates high (red) and low (black) expression. The responsive transcripts are listed on the right of panel. We used the heatmap.2 in the R package gplots (ver. 2.11.0) to generate heat maps with the Z-scores of RPKM values. Click here for file

Additional file 5: Figure S3 Differential gene expression heat map from Table 3. Z scores of RPKM values for each sample were shown in heat map. The bar in red-black gradation indicates high (red) and low (black) expression. The responsive transcripts are listed on the right of the panel. Click here for file

Additional file 6: Figure S4 Differential gene expression heat map from Table 4. Z scores of RPKM values for each sample were shown in heat map. The bar in red-black gradation indicates high (red) and low (black) expression. The responsive transcripts are listed on the right. A, These genes are little affected by K833A mutation of OsIRE1 (We called ‘Type (1)’ in text). B, These genes are moderately affected by K833A mutation of OsIRE1 (We called ‘Type (2)’ in text). C, These genes are drastically affected by K833A mutation of OsIRE1 (We called ‘Type (3)’ in text). Click here for file

Additional file 7: Table S3 Original gene list of the basis of Table 4. Click here for file

Additional file 8: Table S4 List of transcripts exhibiting RIDD-like changes in expression under ER stress conditions. Click here for file

Additional file 9: Table S5 Prediction of micro(mi)RNA target gene. Website [miRbase (http://plantgrn.noble.org/psRNATarget/)] was used for this prediction. Click here for file

Additional file 10: Table S6 Primer sets used for qRT-PCR. Click here for file
