# Supplementary figures and images for: RNA sequencing-mediated transcriptome analysis of rice plants in endoplasmic reticulum stress conditions
Source: BMC Plant Biol. 2014 Apr 18;14:101. doi: 10.1186/1471-2229-14-101 (PMC4021347; doi:10.1186/1471-2229-14-101)

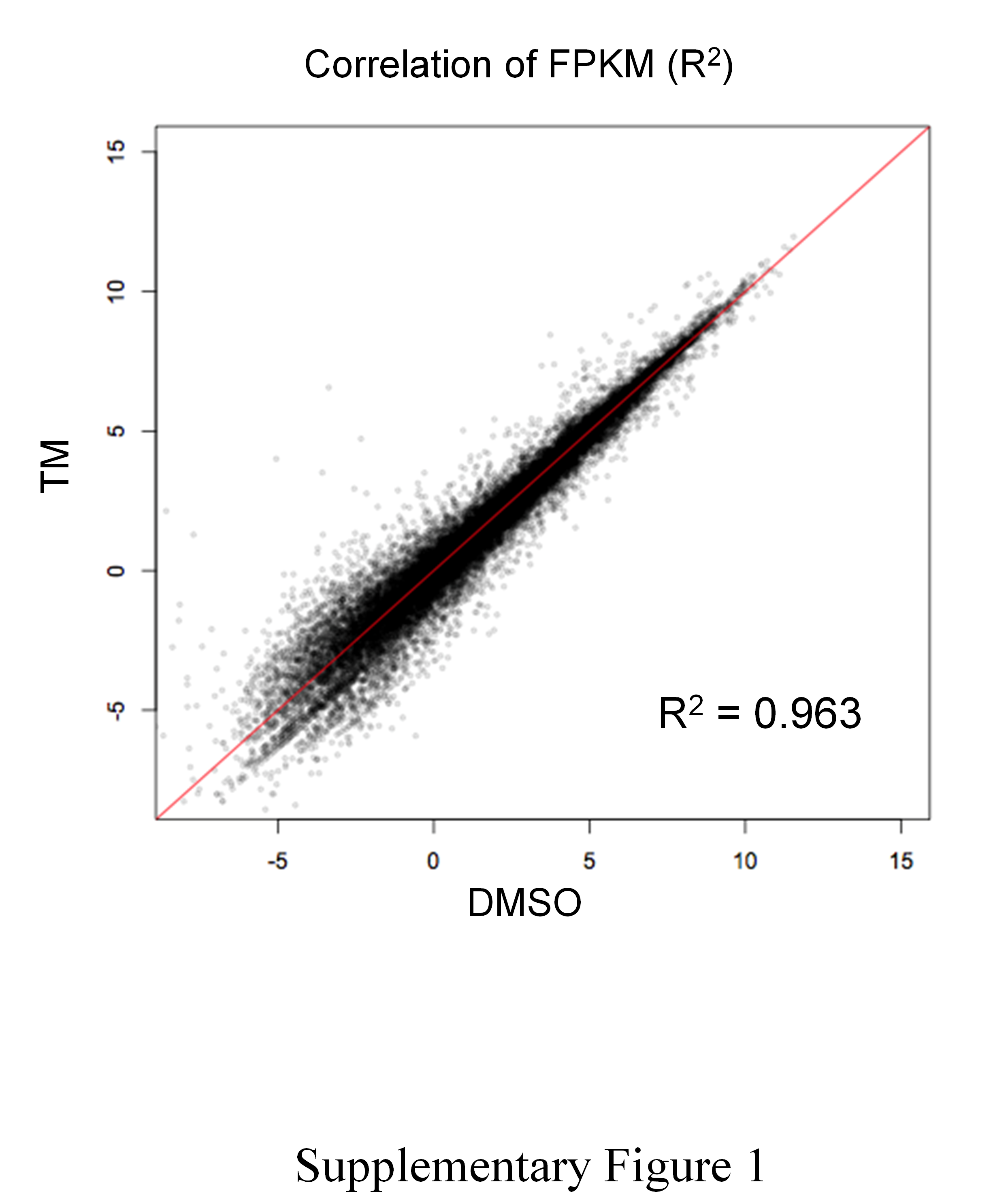

Supplement: Additional file 1: Figure S1 — Quantification of gene expression levels by RNA-Seq analysis in rice roots under TM treatment. Scatter plot shows FPKM (Fragments Per Kilobase of transcript per Million fragments sequenced) values of RNA-Seq data from wild type treated with DMSO and WT treated with TM. The abscissa and ordinate show the FPKM of each treatment. [file 1471-2229-14-101-S1.tiff]

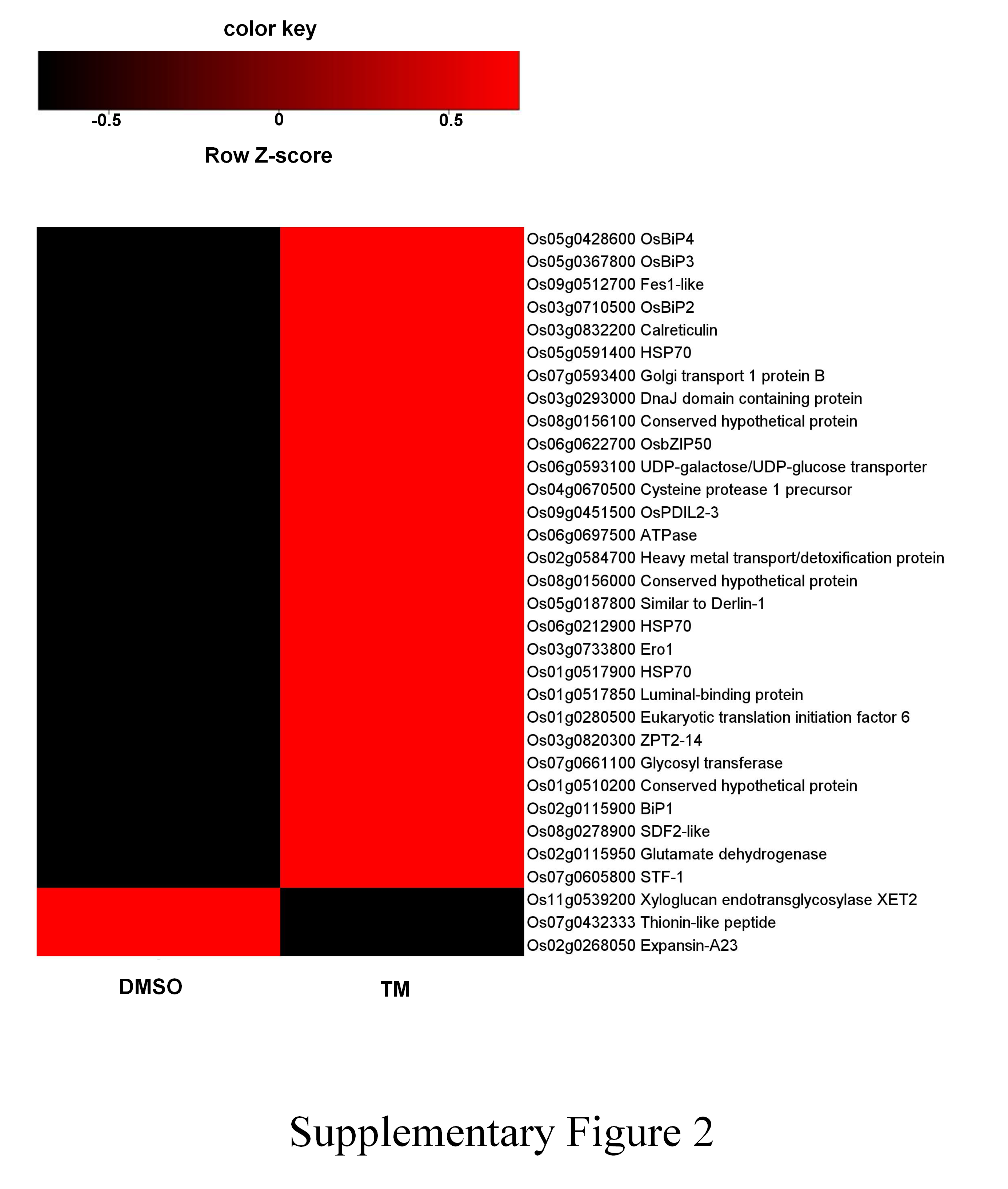

Supplement: Additional file 4: Figure S2 — Differential gene expression heat map from Table 2. Z scores of RPKM (Reads Per Kilobase of exon Model per million mapped reads) values for each sample were shown in heatmap. The bar in red-black gradation indicates high (red) and low (black) expression. The responsive transcripts are listed on the right of panel. We used the heatmap.2 in the R package gplots (ver. 2.11.0) to generate heat maps with the Z-scores of RPKM values. [file 1471-2229-14-101-S4.tiff]

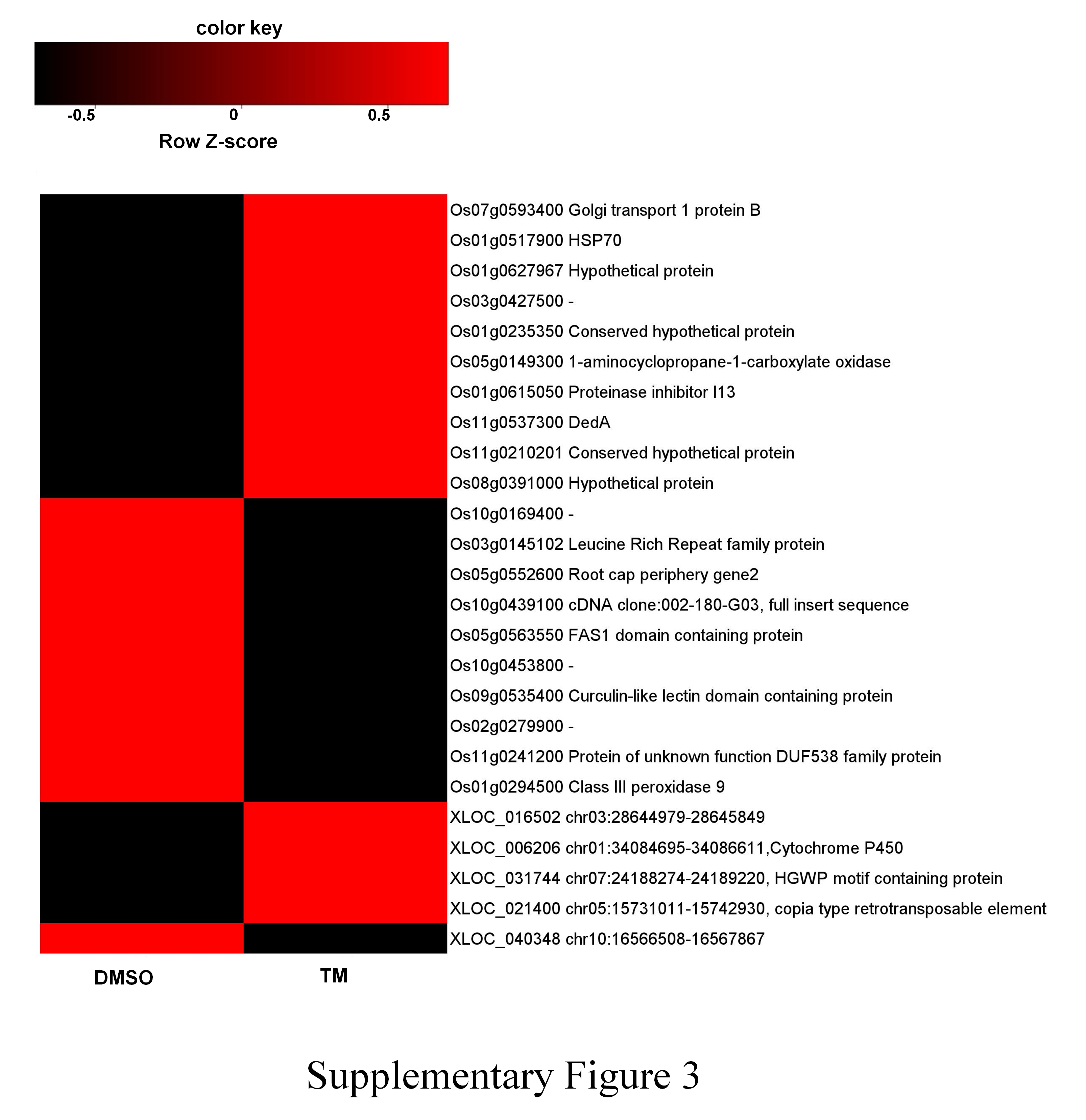

Supplement: Additional file 5: Figure S3 — Differential gene expression heat map from Table 3. Z scores of RPKM values for each sample were shown in heat map. The bar in red-black gradation indicates high (red) and low (black) expression. The responsive transcripts are listed on the right of the panel. [file 1471-2229-14-101-S5.tiff]

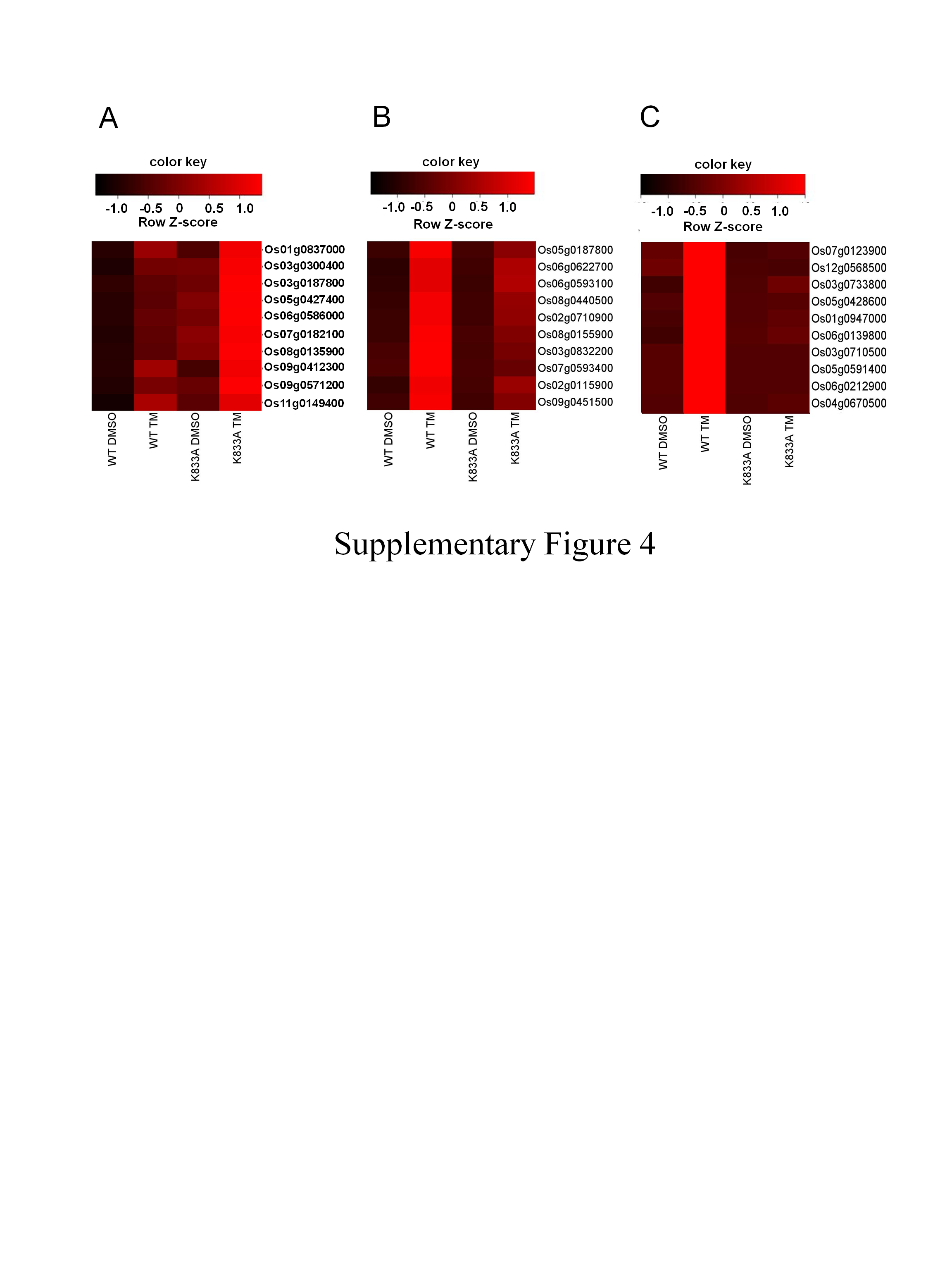

Supplement: Additional file 6: Figure S4 — Differential gene expression heat map from Table 4. Z scores of RPKM values for each sample were shown in heat map. The bar in red-black gradation indicates high (red) and low (black) expression. The responsive transcripts are listed on the right. A, These genes are little affected by K833A mutation of OsIRE1 (We called ‘Type (1)’ in text). B, These genes are moderately affected by K833A mutation of OsIRE1 (We called ‘Type (2)’ in text). C, These genes are drastically affected by K833A mutation of OsIRE1 (We called ‘Type (3)’ in text). [file 1471-2229-14-101-S6.tiff]
